# Celery

**Published:** 1891-12

**Authors:** 


### CELERY.

Probably no class of people suffer more with rheumatism than farmers, and yet the remedy for this dreadful disease is, or should be, right at hand. If celery were eaten freely, sufferers from rheumatism would be comparatively few. It is a mistaken idea that cold and damp produce the disease; they simply develop it. Acid blood is the primary and sustaining cause. If celery is eaten largely, an alkaline blood is the result, and where this exists there can be neither rheumatism nor gout. It should be eaten cooked. Cut it into bits, and boil till soft, in as little water as possible. Add to this half as much milk as there is water in the celery, thicken with flour, and season with butter, pepper, and salt. If you cook it nicely and give it a fair trial, I am sure you will as soon leave potatoes out of the daily bill of fare as celery. It is nice as sauce for any kind of cold meat or fowl, or for roasted poultry or game of any kind. Children will like it poured over boiled potatoes, or it may be drained from the sauce, mixed with mashed potatoes, formed into little cakes and browned. A ready witted woman will find numerous ways of using it.

